# This house believes the NHS should be privatised — 1st southwest medical debate

**DOI:** 10.1186/s13010-015-0031-z

**Published:** 2015-09-22

**Authors:** K. Naguleswaran, T. Tribedi, J. Fenn, S.B. Patel, Peninsula College of Medicine and Dentistry

**Affiliations:** Plymouth University Peninsula Schools of Medicine and Dentistry, Plymouth, UK; Peninsula College of Medicine and Dentistry, Exeter, UK; Peninsula Medical School, Royal Devon and Exeter Hospital, Exeter, UK

## Abstract

The inaugural southwest medical debate, between Exeter and Plymouth medical schools and respective health services, was held on the 3rd December 2014. Plymouth proposed the motion “This house believes the NHS should be privatised?” In an increasingly political climate, the National Health Service (NHS) has become a constant topic for discussion in the media. On this occasion, all those debating were involved in the medical profession with roles encompassing clinical medicine, education, ethics, economics and policy. By allowing those with knowledge of the NHS to speak, we hoped to spark novel discussions based on evidence and experience.

## Background

The post-war Labour government created a platform for hospitals, clinicians, nurses, pharmacists, dentists and opticians to come together in the form a national healthcare service (NHS) that remains free at the point of need. This central principle has featured in many political campaigns, with each political party claiming that the NHS would be ‘safe’ from privatisation in the hands of their government. Though the NHS has always been a mixed system, composed of both public and private provisions, the Labour government of the early 2000s’ created reforms in which the private sector became more involved in the delivery of healthcare. The expansion of the private sector into the NHS was further catalysed by the passing of the Health and Social Care Act in 2012 [1]. With the current political climate and upcoming government elections, the debate on privatization of the NHS could not be any more contemporary.

## Avenues of privatisation

Plymouth began the debate by presenting several ways in which successful privatisation had already occurred both in the UK health services and amongst systems which could be imported to the UK. The first of these privatisation avenues was the current shape of general practice. Plymouth described such practises as private partnerships owned by the general practitioners who work in them. Plymouth raised the topic of the recent Commonwealth Fund report that ranked the NHS as the number one health care system compared to ten other healthcare systems [2]. The sectors, which the NHS scored particularly highly, were patient communication, management of chronic disease and continuity of care, all of which could be accredited to GPs (a private aspect of the health system). Negatively reviewed aspects included hospital acquired infections and unplanned readmissions, both of which are accredited to hospitals (a public section of the health system). Plymouth built on the Commonwealth Fund report by discussing methods of hospital privatisation abroad, stating the most successful hospitals in the USA and Germany are charity or scientific institutes. A 2003 BMJ article on Kaiser Permanente was mentioned, demonstrating the average NHS bed day usage being three and a half times that of a Californian hospital using the aforementioned medical insurance [3]. Hence, Plymouth stated, privatisation has been proven to reduce the amount of time patients spend in hospital.

Plymouth’s next avenue to privatisation was patient owned co-operatives in healthcare, starting by making reference to the Seattle based healthcare co-operative Group Health as a successful example of privatised health care [4]. Group Health boasts a board of trustees comprised of patients, complete vertical integration of services, and coverage of 600,000 patients in the Washington and Idaho areas making it an attractive model for importation to the NHS. To further the suggestion of co-operatives, Plymouth made reference to the fact that already many of GP’s out of hours services are provided by patient owned co-operative enterprises [5]. As mentioned previously, this area of the NHS was the portion providing a majority of indicators used to evaluate the NHS as first ranked in the Commonwealth fund report [2].

## Rankings, ratings and reports

Exeter opened the case for the opposition by presenting various different tenants that encompassed ethics, professional codes of conduct, efficiency and accessibility. Drawing upon personal experiences, Exeter reminded us of the life before a nationalised service. A life in which healthcare was a commodity. The opposition’s argument then proceeded to explain that privatizing the NHS would effectively dismiss the notion of evidence based medicine that the United Kingdom takes pride in. The evidence from the US, Africa, and Republic of Ireland have all unanimously shown that that making the patient pay will only deter the most socio-economically deprived from accessing healthcare services. Substantiating this point further, two comparison studies of mortality rates in profit and non-profit hospitals were presented. There systemic reviews revealed that the mortality rates of for-profit hospitals were higher [6]. Therefore, Exeter argued, privatisation has failed the ultimate test of providing for the most vulnerable. The 2014 release of the Commonwealth Fund rankings were central to Exeter’s case on the efficiency of the NHS. Even with the lowest health expenditure per capita of $3405, the NHS ranked number one [2]. Meanwhile, the privatised system of the US, spending an impressive $8508 on health expenditure per capita, fared rather poorly in the overall rankings. Despite spending twice as much gross domestic product on healthcare as the UK, the US had worse infant mortality rates and lower life expectancy. By using the US as a prime example, Exeter established the failures of privatisation.

Exeter went on to highlight the perks of a nationalised system such as the temporary residency scheme, which would not be possible if the NHS was privatised. Schemes such as this illustrated how the NHS could prioritise ethical considerations above economic consequences. Exeter closed their opening argument by outlining the consequences of privatisation through the means of an anecdotal example from a general practice in Cornwall. A virtually complaint-free practice, run by local general practitioners, underwent privatisation and the number of negative outcomes escalated dramatically. 252 fraudulent entries, soaring A&E admission (due to poor primary care) and an influx of complaints resulted in such as short of period of time that this specific practise was highlighted in parliament as evidence of the perils of privatisation.

## Stress in the NHS

Plymouth explained that their stance was based on the premise that privatisation of the NHS was not supposed to rework the NHS entirely but intended to solely change the governing. Plymouth focused on the staff of the NHS and their experience of working within the NHS. They described the NHS in terms of being comprised of two equally significant groups: the staffs who lend their expertise to the service in exchange for a livelihood and the patients who utilise this service. Plymouth started by drawing attention to findings that arose from the BMA doctor cohort study, a 10-year study of 431 junior doctors who graduated in 2006. The BMA revealed that 28 % of the junior doctors in the study said they did not have the time to deliver the level of care patients deserved [7]. A further 44 % of junior doctors described their stress levels as progressively worsening over the previous year [7]. In addition, Plymouth emphasised the findings of the Labour Force Survey (LFS) in regards to NHS workers, which showed that the number of staff members who reported suffering severe work related stress rose significantly to 38 % in 2012 from 30 % in 2011 and 29 % in 2010 [8]. These findings supported the claim, by Plymouth, that the NHS needed change by demonstrating the high levels of unnecessary stress amongst those working within. In addition, Plymouth pointed out that the evidence suggested the staff were not managing time well enough to allow delivery of the highest level of care - an integral component of the health service and something that is expected by the general public.

## The miracle of the market

Competition drives innervation and increases cost effectiveness. Plymouth argued the effectiveness of such competition using the evolution of the smartphone as an idealised example. Hence, perhaps a privatised healthcare system would create a similarly novel market, streamlining the service into a more efficient provider? Exeter rebutted this notion by employing basic economic theory. In order for a flourishing market to occur, the consumer requires an adequate amount of information about the options available. With a privatised healthcare system, the multitude of choices available could make it difficult for the patient to make an informed decision. Hence, patients’ health could be detrimentally affected by opening the market. On a more ethical basis, Exeter created discomfort by describing the prospects of capitalising on a vulnerable individual’s ill health and suffering. Not only did this directly conflict with the tenants of medical ethics (such as beneficence, autonomy, justice and non-maleficence) but also chillingly prioritised the economic success of the health service over all others.

## Shortfalls in equality

Plymouth referred back to the Commonwealth Fund report comparing health services, first examining why it was that the NHS scored so highly. The NHS scored highly on effective care, which is based upon factors such as the proportion of hypertensive patients who receive the recommended frequency of blood pressure reviews. Plymouth suggested that it was no surprise that the NHS was high scoring in those activities, as they are incentivised by the Quality and Outcome Frameworks (QOF) [9]. Next, focusing in on the rank the UK received for health outcomes, Plymouth pointed out the UK ranked at tenth (the US at eleventh only scored lower) in this area [2]. This was accredited to many contributing features all of which could be linked back to Plymouth’s previous comments on indicators that the NHS required management change. The first of these features was the political nature of the NHS; there is no separation of central national government and the health service. This put the health service in a position in which it is vulnerable to political influence, such as the shifting of services and resources to the constituencies of the current party in power. This would not be tolerated or possible in other European countries due to a separation of health services from the central government. Another factor Plymouth highlighted was the idea of a ‘postcode lottery’ for healthcare where by we no longer had a truly universal health system. What the NHS had produced was a large inequality in services. This was due to a combination of political manipulation and distribution of resources being mismatched with the amount of medical care needed.

Plymouth led on from the shortfalls of the NHS by examining the widely used Europe model of social insurance, implying this was a viable direction for privatisation of the NHS [10]. The countries with this model of healthcare were stated to have lower waiting times, more beds available and lower numbers of health benefit receivers compared to the UK. These statistics, Plymouth argued, were a consequence of health services having to provide the health benefits along with the health insurance. A conscious drive to innovate and provide high services, in an attempt to get patients back to work and ultimately reduce cost, resulted from a system of social insurance.

## Closing remarks

Exeter connoted privatisation to a system similar to that of the US, ‘voluntary insurance’ model [10]. Plymouth clarified that they were not an advocate for such a system and proceeded to define their support of a health service that gave universal coverage, ensured a high level of quality regardless of ability to pay and provided the best health outcomes with the greatest efficiency. Plymouth also expanded to say that the core services of a health system should not be provided by ‘for profit’ companies.

At this point Exeter contest the definition of the motion by alluding to the idea that the prototype proposed by Plymouth was not really one of privatisation. Exeter went on to conclude through the words of Richard Titmuss: “Economists may fragment systems and values; other people do not” [11]. In encouraging us to be the ‘other people’, Exeter raised concerns for the future of a society in which health care becomes a commodity: a society that has deviated from philanthropy.

Plymouth closed with a final statement emphasising to doctors and medical students that with the current system they will have fewer resources, less autonomy and even lower pay compared to that of their European colleagues. Exeter closed by defending the NHS, citing criticisms put forward by Plymouth were due to the partial privatisation. Complete privatisation, therefore, would only exacerbate the situation.

## Conclusion

The judges and an audience swing vote awarded the victory to Exeter. The judges stated that Exeter had struck a cord by using ethical arguments and founding principles of the NHS to convey the negative implications of privatisation. In the end, the arguments based on prioritising patient-centred health care over economic progress were unanimously more popular.

Both teams were praised for making passionate speeches and coming up with novel arguments to illustrate differing points of view. The audience, which comprised of a health mix of medical practitioners and students, were treated to a debate filled with interesting perspectives and respectful rhetoric – something missing from the politically driven NHS campaigns in the media.
